# Instrumentation bias in the use and evaluation of scientific software: recommendations for reproducible practices in the computational sciences

**DOI:** 10.3389/fnins.2013.00162

**Published:** 2013-09-09

**Authors:** Nicholas J. Tustison, Hans J. Johnson, Torsten Rohlfing, Arno Klein, Satrajit S. Ghosh, Luis Ibanez, Brian B. Avants

**Affiliations:** ^1^Department of Radiology and Medical Imaging, University of VirginiaCharlottesville, VA, USA; ^2^Department of Psychiatry, University of IowaIowa City, IA, USA; ^3^SRI InternationalMenlo Park, CA, USA; ^4^Department of Psychiatry and Behavioral Science, Stony Brook University School of MedicineStony Brook, NY, USA; ^5^Massachusetts Institute of Technology, McGovern Institute for Brain ResearchCambridge, MA, USA; ^6^Kitware Inc.Clifton Park, NY, USA; ^7^Penn Image Computing and Science Laboratory, Department of Radiology, University of PennsylvaniaPhiladelphia, PA, USA

**Keywords:** best practices, open science, comparative evaluations, confirmation bias, reproducibility

## 1. Introduction

By honest I don't mean that you only tell what's true. But you make clear the entire situation. You make clear all the information that is required for somebody else who is intelligent to make up their mind.

Richard Feynman

The neuroscience community significantly benefits from the proliferation of imaging-related analysis software packages. Established packages such as SPM (Ashburner, 2012), the FMRIB Software Library (FSL) (Jenkinson et al., 2012), Freesurfer (Fischl, 2012), Slicer (Fedorov et al., 2012), and the AFNI toolkit (Cox, 2012) aid neuroimaging researchers around the world in performing complex analyses as part of ongoing neuroscience research. In conjunction with distributing robust software tools, neuroimaging packages also continue to incorporate algorithmic innovation for improvement in analysis tools.

As fellow scientists who actively participate in neuroscience research through our contributions to the Insight Toolkit^1^ (e.g., Johnson et al., 2007; Ibanez et al., 2009; Tustison and Avants, 2012) and other packages such as MindBoggle,^2^ Nipype^3^ (Gorgolewski et al., 2011), and the Advanced Normalization Tools (ANTs),^4^ (Avants et al., 2010, 2011) we notice an increasing number of publications that intend a fair comparison of algorithms which, in principle, is a good thing. Our concern is the lack of detail with which these comparisons are often presented and the corresponding possibility of *instrumentation bias* (Sackett, 1979) where “defects in the calibration or maintenance of measurement instruments may lead to systematic deviations from true values” (considering software as a type of instrument requiring proper “calibration” and “maintenance” for accurate measurements). Based on our experience (including our own mistakes), we propose a preliminary set of guidelines that seek to minimize such bias with the understanding that the discussion will require a more comprehensive response from the larger neuroscience community. Our intent is to raise awareness in both authors and reviewers to issues that arise when comparing quantitative algorithms. Although herein we focus largely on image registration, these recommendations are relevant for other application areas in biologically-focused computational image analysis, and for reproducible computational science in general. This commentary complements recent papers that highlight statistical bias (Kriegeskorte et al., 2009; Vul and Pashler, 2012), bias induced by registration metrics (Tustison et al., 2012), and registration strategy (Yushkevich et al., 2010) and guideline papers for software development (Prlic and Procter, 2012).

## 2. Guidelines

A comparative analysis paper's longevity and impact on future scientific explorations is directly related to the completeness of the evaluation. A complete evaluation requires preparation (before any experiment is performed) and effort to publish its details and results. Here, we suggest general guidelines for both of these steps most of which derive from basic scientific principles of clarity and reproducibility.

### 2.1. Designing the evaluation study

The very idea that one (e.g., registration) algorithm could perform better than all other algorithms on all types of data is fundamentally flawed. Indeed, the “No Free Lunch Theorem” provides bounds on solution quality. That is, it specifically demonstrates that “improvement of performance in problem-solving hinges on using prior information to match procedures to problems” (Wolpert and Macready, 1997). Therefore, the first thing that authors of new algorithms should do is identify how their methods differ with respect to other available techniques in terms of the use of prior knowledge. Furthermore, the author must consider if it is possible to incorporate prior knowledge across existing methods.

#### 2.1.1. Demand that the algorithm developers provide default parameters for the comparative context being investigated

Expert knowledge of a specific program and/or algorithm is most likely found with the original developers who would be in a position to provide optimal parameterization. Relevant parameter files and sample scripts that detail command line calls should accompany an algorithm to aid in its proper use, evaluation, and comparison. For example, the developers of the image registration program elastix (Klein et al., 2010) provide an assortment of parameter files on a designated wiki page^5^ listed in tabular format complete with short description (including applied modality and object of interest) and any publications which used that specific parameter file. Another example is the National Alliance for Medical Image Computing registration use case inventory^6^ where each listed case comprises a test dataset, a guided step-by-step tutorial, the solution, and a custom Registration Parameter Presets file with optimized registration parameters.

#### 2.1.2. Do not implement your own version of an algorithm, particularly if one is available from the original authors. if you must re-implement, consider making your implementation available

Much is left unstated in published manuscripts where novel algorithmic concepts are presented. Ideally, the authors provide an instantiation of the code to accompany the manuscript. As observed in Kovacevic (2006), however, this is often not the case (even in terms of pseudocode). As a result, comparative evaluations are sometimes carried out using code developed not by the original authors but by the group doing the comparison. For example, in Clarkson et al. (2011), the authors compared three algorithms for estimating cortical thickness. Two of the algorithms were coded by the authors of the study while the third was used “off the shelf.” Thus, a natural question to ask is whether the performance difference is due to the algorithm itself, implementation quality, and/or the parameter tuning. None of these are addressed by Clarkson et al. (2011) which may decrease the publication's usefulness.

#### 2.1.3. Perform comparisons on publicly available data

For reasons of reproducibility and transparency, evaluations should be performed using publicly available data sets. Given the rather large number of such institutional efforts including NIREP,^7^ IXI,^8^ NKI,^9^ OASIS,^10^ Kirby,^11^ LONI,^12^ and others, evaluations should include (if not be exhausted by) comparisons using such data. While evaluation on private cohorts might exclude such possibilities, such evaluations should be extensively motivated in the introduction and/or discussion. For example, if a particular algorithm with general application is found to perform better on a private cohort of Parkinson's disease subject data, reasons for performance disparity should be offered and supplemented with analysis on public data.

### 2.2. Publishing the evaluation

#### 2.2.1. Include parameters

In Klein et al. (2009), 14 non-linear registration algorithms were compared using four publicly available, labeled brain MRI data sets. As part of the study, the respective algorithms' authors were given an opportunity to tune the parameters to ensure good performance which were then distributed on Prof. Klein's website.^13^ In contrast, not specifying parameters leaves one susceptible to criticisms of confirmation and/or instrumentation bias. For example, in a recent paper (Haegelen et al., 2013),^14^ the authors compared their ANIMAL registration algorithm with SyN (Avants et al., 2011) and determined that “registration with ANIMAL was better than with SyN for the left thalamus” in a cohort of Parkinson's disease patients. The difference in the authors' experience and investment between the two algorithms could bias algorithmic performance assessment. However, inclusion of parameter settings for ANIMAL and SyN would permit independent verification by reviewers or readers of the article.

#### 2.2.2. Provide details as to the source of the algorithm

Origin should be provided for any code or package used during the evaluation. For example, N4 (Tustison et al., 2010) is a well-known inhomogeneity correction algorithm for MRI first made available as a tech report (Tustison and Gee, 2009). However, since its inclusion in the Insight Toolkit, different programs have been made available. N4 is also available in ANTs (the only version directly maintained by the original authors), as a module in Slicer,^15^ a wrapper of the Slicer module in Nipype,^16^ a module in c3d,^17^ and as a plugin in the BRAINS suite.^18^ While each version is dependent on the original source code, there could exist subtle variations which can affect performance. As one specific example, the c3d implementation hard-codes certain parameter values with no access to modify them by the user.

#### 2.2.3. Co-authors should verify findings

Although different journals have varying guidelines for determining co-authorship, there is at least an implied sense of responsibility for an article's contents assumed by each of the co-authors. Strategies taken by journal editorial boards are used to reduce undeserving authorship attribution such as requiring the listing of the specific contributions of each co-author. Additional proposals have included signed statements of responsibility for the contents of an article (Anonymous, 2007). We suggest that at least one co-author independently verify a subset of the results by running the data processing and analysis on their own computational platform. The point of this exercise is to verify not only reproducibility but also that the process can be explained in sufficient detail.

#### 2.2.4. Provide computational platform details of the evaluation

A recent article (Gronenschild et al., 2012) pointed out significant differences in FreeSurfer output that varied with release version and with operating system. While the former is to be expected given upgrades and bug fixes which occur between releases, the latter underscores both the need for consistency in study processing as well as the reporting of computational details for reproducibility.

#### 2.2.5. Supply pre- and post-processing steps

In addition to disclosure of all parameters associated with the methodologies to be compared, all processing steps from the raw to the final processed images in the workflow need to be specified. Tools like Nipype (Gorgolewski et al., 2011) capture this provenance information in a formal and rigorous way, but at a minimum the shell scripts or screen shots of the parameter choices should be made available. Justification for any deviation of steps between algorithms needs to be provided.

#### 2.2.6. Post the resulting data online

The current publishing paradigm limits the quantity of results that can be posted. There are only so many pages allowed for a particular publication and displaying every slice of every processed image, for example, is not feasible. This results in possible selection bias where results provided in the manuscript are selected by the authors for demonstrating the effect postulated at the onset of the study. Thus, differences in performance assessment tend to be exaggerated based strictly on visual representations in the paper. Publication simply in print (or as figures in a PDF file) and its limitations in terms of dynamic range or spatial resolution also severely limits the ability of reviewers and readers to perform more sophisticated evaluation beyond simple visual inspection.

Alternatively (or additionally), online resources such as the the LONI Segmentation Validation Engine (Shattuck et al., 2009)^19^ can be used to evaluate individual algorithms for brain segmentation on publicly available data sets and compare with previously posted results. A top ranking outcome provides significant external validation for publishing newly proposed methodologies (e.g., Eskildsen et al., 2012).

#### 2.2.7. Put comparisons and observed performance differences into context

In addition to algorithmic and study specifics, it is important to discuss potential limitations concerning qualitative and/or quantitative assessment metrics. In Rohlfing (2012), the author pointed out deficiencies in using standard overlap measures and image similarity metrics in quantifying performance of image registration methods. Other issues, such as biological plausibility of the resulting transforms, need to also be considered. Also important for inclusion is discussion of the possible reasons for performance disparity. If one algorithm outperforms another, reporting of those findings would be much more significant if the authors discuss possible reasons for relative performance levels.

## 3. Conclusion

Considering that computational sciences permeate neuroimaging research, certain safeguards should be in place to prevent (or at least minimize) potential biases and errors that can unknowingly affect study outcomes. There is no vetting agency for ensuring that analysis programs used for research are reasonably error-free. In addition, these software packages are simply “black boxes” to many researchers who are not formally trained to debug code, and who, in most cases, have only a very superficial understanding of the algorithms that they apply. And even to those of us who are trained to debug code, understanding someone else's code, perhaps implemented in an unfamiliar programming language and different coding style, is oftentimes very difficult. To this end, algorithmic comparisons are a very good way of evaluating general performance. We hope that the guidelines proposed in this editorial help the community in future comparative assessments and avoid errors in scientific computing that may otherwise lead to publication of invalid results (Merali, 2010).

## Footnotes

^1^http://www.itk.org

^2^http://www.mindboggle.info

^3^http://nipy.org/nipype

^4^http://stnava.github.io/ANTs/

^5^http://elastix.bigr.nl/wiki/index.php/Parameter_file_database

^6^http://www.na-mic.org/Wiki/index.php/Projects:RegistrationDocumentation:UseCaseInventory

^7^http://www.nirep.org

^8^http://www.brain-development.org

^9^http://fcon_1000.projects.nitrc.org/indipro/nki.html

^10^http://www.oasis-brains.org

^11^http://mri.kennedykrieger.org/databases.html

^12^http://www.loni.ucla.edu/Research/Databases/

^13^http://mindboggle.info/papers/evaluation_NeuroImage2009.php

^14^Similar issues can be found in Wu et al. (2013).

^15^http://www.slicer.org/slicerWiki/index.php/Documentation/4.2/Modules/N4ITKBiasFieldCorrection

^16^http://www.mit.edu/~satra/nipype-nightly/interfaces/generated/nipype.interfaces.slicer.filtering.n4itkbiasfieldcorrection.html

^17^http://www.itksnap.org8/pmwiki/pmwiki.php?n=Convert3D.Documentation

^18^http://www.nitrc.org/plugins/mwiki/index.php/brains:N4ITK

^19^http://sve.loni.ucla.edu
